# Molecular Mechanism of Jinchan Oral Liquid in the Treatment of Children with Respiratory Syncytial Virus Pneumonia Based on Network Pharmacology and Molecular Docking Technology

**DOI:** 10.1155/2021/6471400

**Published:** 2021-08-26

**Authors:** Li Shen, Yiguo Jiang, Jinmiao Lu, Guangfei Wang, Xiaolan Zhang, Sumei He, Cheng Wang, Zhiping Li

**Affiliations:** ^1^Department of Pharmacy, The Affiliated Suzhou Science & Technology Town Hospital of Nanjing Medical University, Suzhou Jiangsu 215153, China; ^2^Department of Clinical Pharmacy, Children's Hospital of Fudan University, National Children's Medical Center, Shanghai 201102, China; ^3^The Health Supervision Institute of Suzhou High-Tech Zone, Suzhou Jiangsu 215007, China

## Abstract

**Objective:**

Exploration of the underlying molecular mechanism of Jinchan Oral Liquid (JOL) in treating children with the respiratory syncytial virus (RSV) pneumonia to provide new evidence for the clinical application.

**Methods:**

The active components and target genes of JOL were screened by the TCMSP database. The targets of RSV pneumonia were obtained from the GeneCards, OMIM, DrugBank, and PharmGKB database. Then, we constructed the active component-target network and screened the core genes. The overlaps were screened for PPI network analysis, GO analysis, and KEGG analysis. Finally, result validation was performed by molecular docking.

**Results:**

According to the screening criteria of the ADME, 74 active compounds of JOL were obtained; after removing redundant targets, we selected 180 potential targets. By screening the online database, 893 RSV pneumonia-related targets were obtained. A total of 82 overlapping genes were chosen by looking for the intersection. The STRING online database was used to acquire PPI relationships, and 16 core genes were obtained. GO and KEGG analyses showed that the main pathways of JOL in treating RSV pneumonia include TNF signaling pathway and IL17 signaling pathway. The molecular docking results showed that the active compounds of JOL had a good affinity with the core genes.

**Conclusion:**

In this study, we preliminarily discussed the main active ingredients, related targets, and pathways of JOL and predicted the pharmacodynamic basis and the potential therapeutic mechanisms of RSV pneumonia. In summary, the network pharmacology strategy may be helpful for the discovery of multitarget drugs against complex diseases.

## 1. Introduction

Community-Acquired Pneumonia (CAP) is a leading cause of hospitalization in children younger than five years [1]. Respiratory syncytial virus (RSV) is the most common viral pathogen, especially in children under two years of age [2, 3]. More than 50 percent of the RSV hospitalizations occurred in China younger than six months, and RSV infection showed significant seasonal patterns [4]. Palivizumab was market-approved by the FDA for immunoprophylaxis for RSV [5]. However, the clinical application was limited because of its high cost. It is of crucial importance to find safe and effective alternative therapies.

Traditional Chinese medicine has a long history of thousands of years. The prominent feature of Chinese medicine treatment is based on syndrome differentiation, and it emphasizes the recovery of overall function. Jinshan Oral Liquid (JOL) is a traditional Chinese compound prescription developed by Children's Hospital Affiliated to Soochow University. JOL comes from “famous doctors and prescriptions.” It is mainly composed of four herbs: honeysuckle (Jin Yin Hua: JYH), Scutellaria baicalensis (Huang Qin: HQ), bupleurum (Chai Hu: CH), and cicada slough (Chan Tui: CT). Our previous study found that JOL is effective and safe in treating children with respiratory syncytial virus (RSV) pneumonia, and it can improve clinical symptoms and shorten the length of hospital stay. However, the mechanisms by which JOL exerts these effects remained unclear.

**Figure 1:**
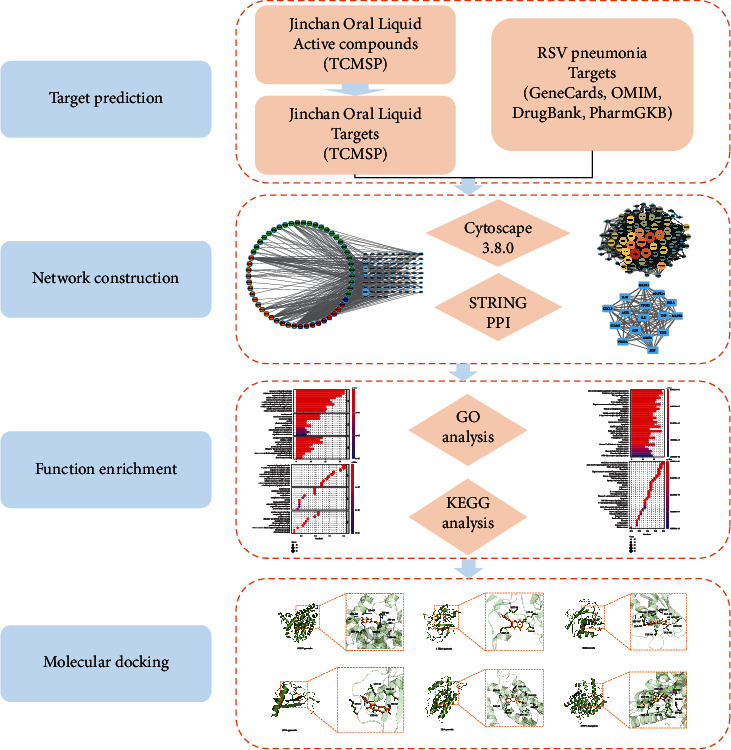
Research process of network pharmacology and molecular docking.

Network pharmacology [6, 7] is a broad discipline based on systems biology and computer technology, which analyzes the interaction network of “disease-gene-target-drug.” In this study, we explored the potential molecular mechanism of JOL in treating RSV pneumonia using network pharmacology and molecular docking. The workflow of our study was shown in Figure 1.

## 2. Methods and Materials

### 2.1. Screening of Active Ingredients and Target Genes

All compounds of the four Chinese medicinal herbs in JOL were collected and integrated by the Traditional Chinese Medicine Systems Pharmacology Database and Analysis Platform (TCMSP, https://tcmspw.com/index.php). TCMSP, a platform of Chinese herbal medicines, captures the relationships between drugs, targets, and diseases [8]. The bioactive components of JOL were selected based on the optimal toxicokinetic ADME rules reported in the literature, oral bioavailability (OB) ≥ 30%, and drug − likeness (DL) ≥ 0.18 [9]. However, the number of active components of cicada slough (CT) was zero according to the above screening criteria. Therefore, we followed the TCMSP User Guide (https://tcmspw.com/load_intro.php?id=43) and adjusted the screening conditions as “OB ≥ 20%” and “DL ≥ 0.1.” The related targets of JOL active ingredients were also obtained from the TCMSP database. Then, the targets were entered into UniProt (https://www.uniprot.org/). Through retrieval and transformation, we finally got the gene symbol of active ingredients.

### 2.2. Disease-Associated Gene Mining

The RSV pneumonia-related target proteins were screened from four sources: (1) the Human Gene Database (GeneCards, https://www.genecards.org/), (2) Online Mendelian Inheritance in Man (OMIM, https://omim.org/), (30 DrugBank database (https://go.drugbank.com/), and (4) Pharmacogenomics Knowledgebase (PharmGKB, https://www.pharmgkb.org/). The keywords “respiratory syncytial virus pneumonia” were used to obtain the disease-associated targets. All targets of RSV pneumonia were gathered together, and the results were visualized by R 3.6.3.

### 2.3. Construction of Active Component-Target Network and Analysis

The active ingredients of JOL and the RSV pneumonia-related targets were input into the Cytoscape v3.8.0 software to construct a compound-target network. Nodes represented the bioactive ingredients or targets in the network, while the connections between the nodes represented the interactions.

### 2.4. Construction of Protein-Protein Interaction (PPI) Network

Overlaps between JOL-related targets and RSV pneumonia-related targets were screened by R 3.6.3 to clarify the interaction between JOL and the disease. Then, the overlapping genes were put into STRING (https://www.string-db.org/) database for PPI analysis. The condition was limited to “Homo sapiens” for species. A high confidence level was set at 0.700 so that we could get the appropriate required interaction score.

### 2.5. Identification of Core Genes and Network Visualization

The CytoNCA plug-in in Cytoscape v3.8.0 was performed for the identification of core genes. According to the topological characteristics of the network, six parameters “Betweenness Centrality (BC),” “Closeness Centrality (CC),” “Degree Centrality (DC),” “Eigenvector Centrality (EC),” “Local Average Connectivity-based method (LAC),” and “Network Centrality (NC)” were selected to screen the core genes. The critical targets for JOL treatment of RSV pneumonia were screened based on the following criteria: BC ≥ Avg(BC), CC ≥ Avg(CC), CC ≥ Avg(DC), EC ≥ Avg(EC), LAC ≥ Avg(LAC), and NC ≥ Avg(NC). The core targets were screened out after being extracted twice.

### 2.6. GO and KEGG Pathway Enrichment Analysis

With the Bioconductor package in R software, Gene Ontology (GO) enrichment analysis and Kyoto Encyclopedia of Genes and Genomes (KEGG) pathway analysis were performed. GO enrichment mainly analyzed the biological process, cellular composition, and molecular function of the targets. KEGG pathway enrichment analyzed the vital biological pathways of the targets.

### 2.7. Molecular Docking

Molecular docking simulation was used to verify the credibility of the study. The structural formula (SDF format) of the active ingredients was downloaded from the PubChem database. Then, we use ChemBioDraw 3D software to create 3-dimensional chemical structures and minimize the energy. The crystal structures of the core genes were obtained from the RCSB PDB database and modified using PyMOL v2.4.0 software, including solvent and organic removal. Before molecular docking, AutoDockTools v1.5.6 software was used to add hydrogen atoms. The core genes were used as receptors, and the active ingredients were used as ligands. AutoDock Vina v1.1.2 was run to perform molecular docking. The online Protein-Ligand Interaction Profiler (PLIP web tool, https://plip-tool.biotec.tu-dresden.de/plip-web/plip/index) was used to analyze the docking results. Finally, PyMOL v2.4.0 software was chosen to visualize the result. The conformation with the best affinity and the lowest binding energy was selected as the final docking result.

## 3. Results

### 3.1. Active Components of Screening for JOL

**Figure 2:**
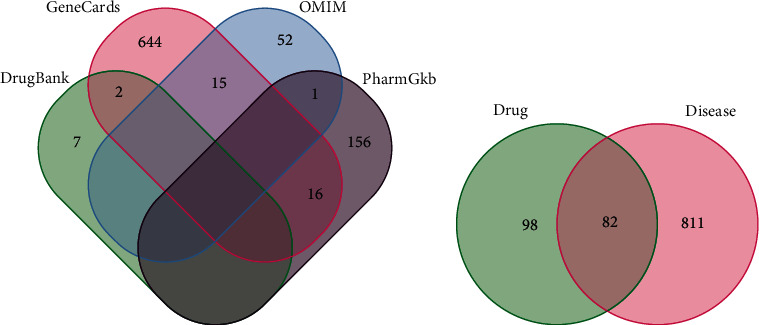
Screening of JOL-RSV pneumonia common targets: (a) Venn diagram of RSV pneumonia-related targets; (b) Venn diagram of drug-disease common targets.

In this study, components of four herbal medicines in JOL were collected, of which Jin Yin Hua (JYH, honeysuckle), Huang Qin (HQ, Scutellaria baicalensis), Chai Hu (CH, bupleurum), and Chan Tui (CT, cicada slough) were identified from the TCMSP database. Based on the screening criteria of the ADME, 74 active compounds were retrieved after duplicated targets were eliminated. These active compounds originated from JYH (23 components), HQ (36 components), CH (17 components), and CT (3 components), including five duplicated targets. Details of the active component information are shown in Supplementary Table 1. All the targets of the active components were predicted by the TCMSP database. After removing redundant ones, 180 potential targets of JOL were finally obtained. By screening the GeneCards database, OMIM database, DrugBank database, and PharmGKB database, 893 RSV pneumonia-related targets were selected (Figure 2(a)). A total of 82 overlapping genes were obtained by looking for the intersection of the above drug targets and the disease targets (Figure 2(b)). These 82 genes were selected as potential targets for further analysis.

### 3.2. Common Active Component-Target Network and Analysis

**Figure 3:**
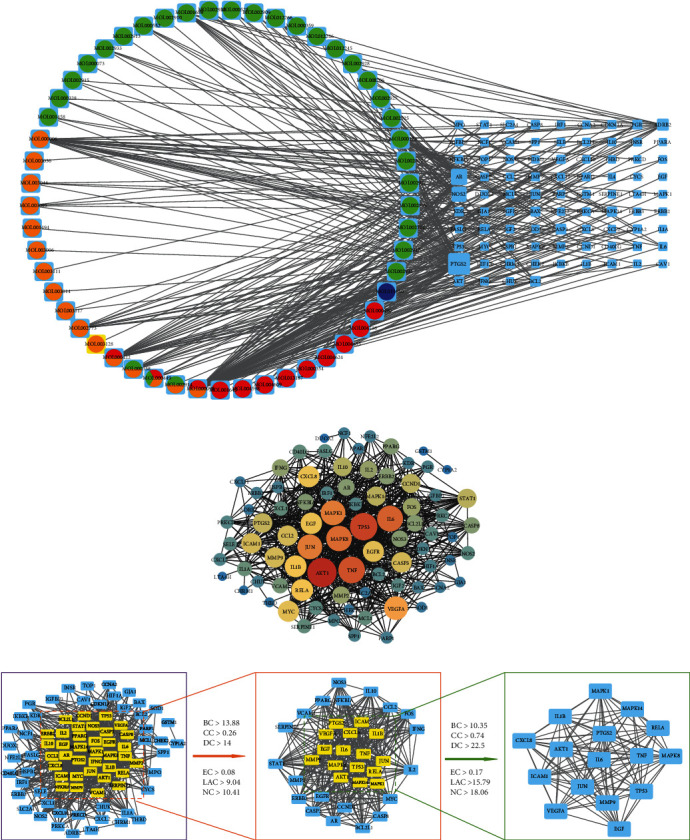
Network construction. (a) Common active component-target network. The circular nodes on the left represent active components, while nodes on the right represent common targets. (b) PPI network of overlapping genes. (c) Identification of core genes.

The active component-target interaction network was constructed by Cytoscape v3.8.0 software (Figure 3(a)). The circular nodes with different colors represented the active components of JOL, and each edge described the relationship between the functional components and target genes. Among those circular nodes, the green ones represented the active components of Scutellaria baicalensis, the orange ones represented the active components of honeysuckle, the red ones represented the active components of bupleurum, and the dark blue ones represented the active components of cicada slough. Based on the degree value, we identified the top three active ingredients, namely, quercetin (MOL000098), luteolin (MOL000006), and kaempferol (MOL000422).

### 3.3. PPI Network Analysis

The STRING online database was used to acquire PPI relationships of potential protein targets of JOL as related to the treatment of RSV pneumonia, and the results are shown in (Figure 3(b)). The PPI network was shown to contain 82 nodes and 1581 edges, with an average node degree of 38.6, an average local clustering coefficient of 0.747, and a PPI enrichment *p* value of <1.0*e*-16. Network nodes represented proteins, and the edges represented protein-protein associations. The thickness of the lines between nodes meant the confidence prediction of the interaction, and the thicker the line, the stronger the interaction relationship between proteins.

### 3.4. Identification of Core Genes and Topological Network Analysis

The CytoNCA plug-in of Cytoscape v3.8.0 software was used to calculate the topology through network analyzer. As shown in Figure 3(c), 82 protein nodes and 758 edges were obtained for intersection genes. After screening with BC > 13.88, CC > 0.26, DC > 14.00, EC > 0.07, LAC > 9.04, and NC > 10.41, the first 36 proteins were obtained, with a total of 397 edges. Then, after second screening with BC > 10.35, CC > 0.74, DC > 22.50, EC > 0.17, LAC > 15.79, and NC > 18.06, the final 16 genes are shown in Table 1 (in descending order of degree) and (Figure 3(c)), with a total of 108 edges. Therefore, we speculated that the 16 core genes encode proteins in pivotal roles.

### 3.5. GO and KEGG Analysis

**Figure 4:**
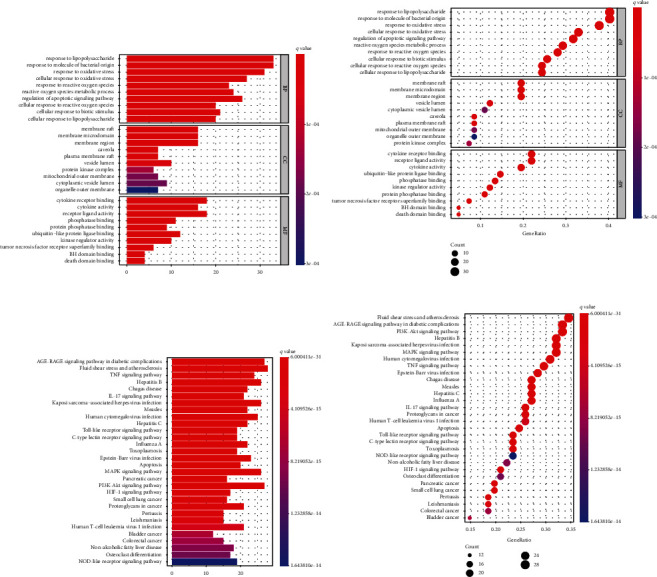
GO and KEGG analysis of drug-disease common genes. (a) Bar plot of GO analysis: top ten significantly enriched terms in BP, CC, and MF, respectively. (b) Bubble chart of GO analysis: the darker the color, the smaller the *q* value. The larger the circle, the more target genes are enriched. (c) Bar plot of KEGG analysis: top 30 significantly enriched terms. (d) Bubble chart of KEGG analysis.

To explore the mechanism of JOL in the treatment of RSV pneumonia, we used R 3.6.3 software to perform GO and KEGG functional enrichment analysis on the 82 drug-disease common genes. GO analysis included three levels: biological process (BP), cellular component (CC), and molecular function (MF). A total of 2475 GO enrichment results were obtained, of which are BP 2305, CC 47, and MF 123. The top ten terms in BP, CC, and MF are, respectively, shown in Figures 4(a) and 4(b). BP mainly involved aspects of response to lipopolysaccharide, response to molecule of bacterial origin, and response to oxidative stress. CC was primarily related to the membrane raft, membrane microdomain, and membrane region. MF was mostly involved in cytokine receptor binding, cytokine activity, and receptor-ligand activity. KEGG pathway analysis was used to determine related signaling pathways associated with the RSV pneumonia of JOL. A total of 155 KEGG enrichment results were selected, and the 30 significant KEGG pathways (*q* value < 0.05) are shown in Figures 4(c) and 4(d). Some played essential roles, including AGE-RAGE signaling pathway in diabetic complications, fluid shear stress and atherosclerosis, TNF signaling pathway, hepatitis B, Chagas disease, IL-17 signaling pathway, and Kaposi sarcoma-associated herpesvirus infection. The potential pathways were mainly enriched in the categories of the inflammatory response, fighting against viruses and other pathogens, signal transduction, and immunologic regulation. Besides, we extracted the relevant pathways as in Table 2.

### 3.6. Validation by Molecular Docking

**Figure 5:**
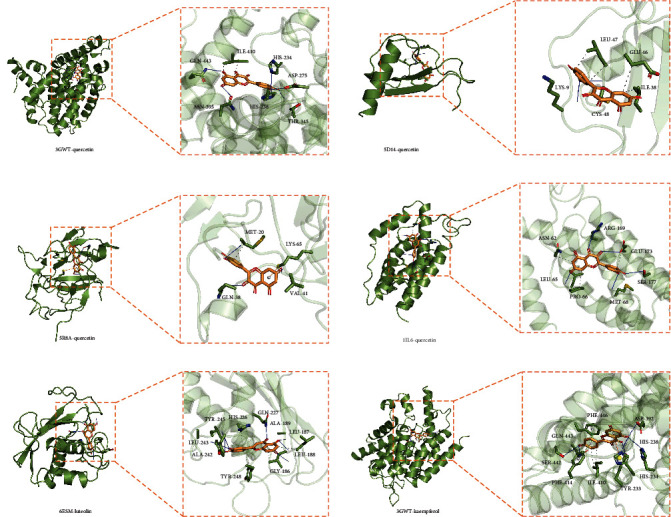
Molecular docking of active compounds and core genes: (a) TNF-quercetin, (b) CXCL8-quercetin, (c) IL-1B-quercetin, (d) IL6-quercetin, (e) MMP9-luteolin, and (f) TNF-kaempferol.

Molecular docking can simulate the interaction between ligand and receptor and predict the affinity by calculating the binding energy. The small-molecule ligand can spontaneously bind to the macromolecular receptor when the binding energy is lower than zero [10]. When the binding energy was lower than -5.0 kcal/mol, the two showed better binding activity [11]. In our study, the five core genes and three active ingredients were used as receptors and ligands. The screening results (Table 3) illustrated that the three active ingredients had a strong affinity with the corresponding protein receptors. Luteolin had the best binding to MMP9 through 6 interactions, mainly including hydrophobic interactions and hydrogen bonds. Interactions between the ligands and the receptors are shown in Figure 5.

## 4. Discussion

As the most common respiratory pathogen, RSV is a cause of morbidity and mortality in those prematurely born infants and children with high-risk factors [12]. Lower respiratory tract infections (LRTI) caused by RSV include bronchiolitis and pneumonia. Studies found that RSV-driven Th2-immunity skewing was closely linked with airway restructuring and asthma during childhood [13, 14]. As a hospital-made preparation, JOL came from “famous doctors and prescriptions” and was widely used in children infected with respiratory viruses. Our previous clinical study found that JOL is effective and safe in treating children with RSV pneumonia. We explored the underlying mechanisms through network pharmacology to provide better clinical evidence.

In the JOL's active component-target network, a total of 180 targets affected by 74 active compounds in the JOL were selected. There were three top important compounds, including quercetin, luteolin, and kaempferol, identified as the potential active ingredients of JOL.

As a natural flavonoid, quercetin showed several biological activities, and it could block the adhesion of RSV [15]. Studies found that quercetin could interact with G protein ectodomain of group A human RSV due to the stable combination between them [16, 17]. It was reported that quercetin could alleviate inflammatory lung injury [18]. Luteolin is a typical flavonoid compound and has been widely studied for its effects, including antioxidant, anticancer, anti-inflammatory, and antiapoptotic [19]. Wang et al. found that luteolin can decrease the titer of RSV and inhibit viral replication [20]. Kaempferol, known as polyphenol, was a flavonol present in different plants. Experimental studies confirmed that kaempferol could inhibit inflammatory cell function by inhibiting the expression of cytokines and chemokines [21, 22].

Then, we retrieved 893 RSV pneumonia targets from GeneCards, OMIM, DrugBank, and PharmGKB database. Through target mapping, we found 82 targets in common between active components and the disease targets and built the connections of the “ingredient-target” network.

By constructing the PPI network, topological parameters for the recognition of essential nodes were calculated. 16 genes were found to be the core genes of JOL in treating RSV pneumonia. These genes were connected with host immunity, oxidative stress, virus, and other pathogenic microorganisms. We focused on the most relevant genes that had previously been reported. Recent studies were performed to evaluate the relative roles of IL-1B, IL-6, TNF-*α*, CXCL8, and MMP9 in acute RSV infection [23–25]. IL6 is a cytokine with multiple biological functions. IL-1B and TNF are primary activators of IL6 expression [26]. The expression of IL6 increased when the host developed with infection, autoimmune disease, or cancer [27]. TNF-*α* played a vital role in RSV-induced exacerbations in allergic airway disease [28]. CXCL8 had been shown to be a chemokine involved in neutrophil activation [29], while RSV infection could increase airway neutrophils [12]. MMP9 is a member of the matrix metalloproteinase family [30]. Expression levels of MMP9 increased during RSV infection of airway epithelia, and it played an essential role in disease severity [25]. Core genes such as TNF, CXCL8, IL-1B, IL-6, and MMP9 were selected to perform molecular docking to verify the interaction between active ingredients and target genes, respectively.

GO and KEGG enrichment analysis revealed that JOL could regulate the progress of immune pathways and virus defense. The ten significant GO (BP) terms indicated that JOL could control the pathogenic microbial stimulation and the oxidative stress process during the treatment of RSV pneumonia. KEGG pathway enrichment results showed potential pathways. Most of the target genes were enriched in the virus-related signaling pathways, TNF signaling pathway, and IL-17 signaling pathway. Several studies [31–33] have demonstrated that the TNF signaling pathway and IL-17 signaling pathway were related to RSV infection. The TNF signaling pathway plays a significant role in immune regulation and inflammation [34]. Experimental evidence demonstrated that TNF receptor blockade could reduce the expression of cytokines and chemokines that closely related to RSV infection [35]. IL-17 signals played an essential role in both transcriptional and posttranscriptional levels [36]. The level of IL-17 increased after suffering from RSV, which was linked to mucus secretion [37].

Serafini et al. reported that flavonoids could inhibit the expression of proinflammatory cytokines/chemokines such as TNF-*α*, IL-6, IL-1B, and IL-8 [38]. Evidence indicated that flavonoids suppress the activation of NF-*κ*B and MAPK, to inhibit the expression of inflammatory cytokines [39]. Based on the GO and KEGG results, our study indirectly showed that the TNF signaling pathway and the IL17 signaling pathway might play a significant role in treating RSV pneumonia with JOL.

We also performed molecular docking to predict complex interactions between the three active ingredients and the protein targets. The binding energies could help to verify the reliability of the docking results further, and the results demonstrated good binding properties. All the binding energy were less than -7 kcal/mol. The interaction points indicated that quercetin, luteolin, and kaempferol might play an important role in treating RSV pneumonia.

## 5. Conclusion

We analyzed the potential mechanism by which JOL effectively treated RSV pneumonia based on network pharmacology. A possible association between active ingredients and core genes was discovered through the systematic strategy, which further verified the reliability by molecular docking technology. Therefore, traditional Chinese medicine such as JOL may be an alternative approach for treating RSV infection. But there were still some limitations. Further experimental validation is needed to support our research.

## Figures and Tables

**Table 1:** Topological analysis results.

Gene names	Betweenness	Closeness	Degree	Eigenvector	LAC	Network
TNF	38.06	0.95	33.00	0.23	20.12	31.65
IL6	35.21	0.95	33.00	0.23	20.55	31.97
JUN	23.88	0.88	30.00	0.22	19.80	27.35
MAPK8	32.28	0.88	30.00	0.21	17.93	25.81
AKT1	33.62	0.88	30.00	0.21	17.60	25.44
TP53	26.79	0.85	29.00	0.20	18.14	25.27
MAPK1	22.26	0.85	29.00	0.21	19.03	25.63
VEGFA	20.42	0.81	27.00	0.19	17.48	22.68
MMP9	17.80	0.80	26.00	0.19	17.31	21.55
IL1B	21.51	0.80	26.00	0.18	16.31	20.73
CXCL8	18.84	0.80	26.00	0.19	17.08	21.32
PTGS2	13.72	0.78	25.00	0.18	17.44	20.90
EGF	14.16	0.78	25.00	0.18	17.12	20.37
RELA	16.32	0.76	24.00	0.17	15.83	19.10
ICAM1	11.38	0.74	23.00	0.17	16.26	19.12
MAPK14	11.09	0.74	23.00	0.17	16.17	18.86

**Table 2:** Relevant pathways enriched by target genes.

ID	Description	GeneRatio	*q* value	Count
hsa04668	TNF signaling pathway	24/81	4.73*e*-25	24
hsa05161	Hepatitis B	26/81	6.24*e*-24	26
hsa04657	IL-17 signaling pathway	21/81	1.83*e*-22	21
hsa05167	Kaposi sarcoma-associated herpesvirus infection	26/81	3.93*e*-22	26
hsa05163	Human cytomegalovirus infection	25/81	3.15*e*-19	25

**Table 3:** Screening docking results between receptors and ligands.

Core genes (PDB ID)	Active ingredients	Binding energy (kcal/mol)
TNF (3GWT)	Quercetin	-9.1
CXCL8 (5D14)	Quercetin	-7.9
IL-1B (5R8A)	Quercetin	-7.6
IL6 (1IL6)	Quercetin	-7.4
MMP9 (6ESM)	Luteolin	-10.8
TNF (3GWT)	Kaempferol	-9.1

## Data Availability

Details of the active component information are shown in Supplementary Table 1. All the data can be downloaded from the open databases mentioned in this article.

## Supplementary Materials

Supplementary Materials Supplementary Table 1: basic information of active components of JOL.
