# Supplementary material for: Molecular Mechanism of Jinchan Oral Liquid in the Treatment of Children with Respiratory Syncytial Virus Pneumonia Based on Network Pharmacology and Molecular Docking Technology
Source: Biomed Res Int. 2021 Aug 26;2021:6471400. doi: 10.1155/2021/6471400 (PMC8416387; doi:10.1155/2021/6471400)
Supplement: Supplementary Materials — Supplementary Table 1: basic information of active components of JOL. [file 6471400.f1.doc]

**Supplementary Table1 Basic information of active components of JOL**

| Mol ID | Chemical component | OB (%) | DL | Herb |
| --- | --- | --- | --- | --- |
| MOL001494 | Mandenol | 42 | 0.19 | honeysuckle |
| MOL001495 | Ethyl linolenate | 46.1 | 0.2 |
| MOL002707 | phytofluene | 43.18 | 0.5 |
| MOL002914 | Eriodyctiol (flavanone) | 41.35 | 0.24 |
| MOL003006 | (-)-(3R,8S,9R,9aS,10aS)-9-ethenyl-8-(beta-D-glucopyranosyloxy)-2,3,9,9a,10,10a-hexahydro-5-oxo-5H,8H-pyrano[4,3-d]oxazolo[3,2-a]pyridine-3-carboxylic acid_qt | 87.47 | 0.23 |  |
| MOL003014 | secologanic dibutylacetal_qt | 53.65 | 0.29 |  |
| MOL002773 | beta-carotene | 37.18 | 0.58 |  |
| MOL003036 | ZINC03978781 | 43.83 | 0.76 |  |
| MOL003044 | Chryseriol | 35.85 | 0.27 |  |
| MOL003059 | kryptoxanthin | 47.25 | 0.57 |  |
| MOL003062 | 4,5'-Retro-.beta.,.beta.-Carotene-3,3'-dione, 4',5'-didehydro- | 31.22 | 0.55 |  |
| MOL003095 | 5-hydroxy-7-methoxy-2-(3,4,5-trimethoxyphenyl)chromone | 51.96 | 0.41 |  |
| MOL003101 | 7-epi-Vogeloside | 46.13 | 0.58 |  |
| MOL003108 | Caeruloside C | 55.64 | 0.73 |  |
| MOL003111 | Centauroside_qt | 55.79 | 0.5 |  |
| MOL003117 | Ioniceracetalides B_qt | 61.19 | 0.19 |  |
| MOL003124 | XYLOSTOSIDINE | 43.17 | 0.64 |  |
| MOL003128 | dinethylsecologanoside | 48.46 | 0.48 |  |
| MOL000358 | beta-sitosterol | 36.91 | 0.75 |  |
| MOL000422 | kaempferol | 41.88 | 0.24 |  |
| MOL000449 | Stigmasterol | 43.83 | 0.76 |  |
| MOL000006 | luteolin | 36.16 | 0.25 |  |
| MOL000098 | quercetin | 46.43 | 0.28 |  |
| MOL001689 | acacetin | 34.97 | 0.24 | scutellaria baicalensis |
| MOL000173 | wogonin | 30.68 | 0.23 |
| MOL000228 | (2R)-7-hydroxy-5-methoxy-2-phenylchroman-4-one | 55.23 | 0.2 |
| MOL002714 | baicalein | 33.52 | 0.21 |
| MOL002908 | 5,8,2'-Trihydroxy-7-methoxyflavone | 37.01 | 0.27 |  |
| MOL002909 | 5,7,2,5-tetrahydroxy-8,6-dimethoxyflavone | 33.82 | 0.45 |  |
| MOL002910 | Carthamidin | 41.15 | 0.24 |  |
| MOL002911 | 2,6,2',4'-tetrahydroxy-6'-methoxychaleone | 69.04 | 0.22 |  |
| MOL002913 | Dihydrobaicalin_qt | 40.04 | 0.21 |  |
| MOL002914 | Eriodyctiol (flavanone) | 41.35 | 0.24 |  |
| MOL002915 | Salvigenin | 49.07 | 0.33 |  |
| MOL002917 | 5,2',6'-Trihydroxy-7,8-dimethoxyflavone | 45.05 | 0.33 |  |
| MOL002925 | 5,7,2',6'-Tetrahydroxyflavone | 37.01 | 0.24 |  |
| MOL002926 | dihydrooroxylin A | 38.72 | 0.23 |  |
| MOL002927 | Skullcapflavone II | 69.51 | 0.44 |  |
| MOL002928 | oroxylin a | 41.37 | 0.23 |  |
| MOL002932 | Panicolin | 76.26 | 0.29 |  |
| MOL002933 | 5,7,4'-Trihydroxy-8-methoxyflavone | 36.56 | 0.27 |  |
| MOL002934 | NEOBAICALEIN | 104.34 | 0.44 |  |
| MOL002937 | DIHYDROOROXYLIN | 66.06 | 0.23 |  |
| MOL000358 | beta-sitosterol | 36.91 | 0.75 |  |
| MOL000359 | sitosterol | 36.91 | 0.75 |  |
| MOL000525 | Norwogonin | 39.4 | 0.21 |  |
| MOL000552 | 5,2'-Dihydroxy-6,7,8-trimethoxyflavone | 31.71 | 0.35 |  |
| MOL000073 | ent-Epicatechin | 48.96 | 0.24 |  |
| MOL000449 | Stigmasterol | 43.83 | 0.76 |  |
| MOL001458 | coptisine | 30.67 | 0.86 |  |
| MOL001490 | bis[(2S)-2-ethylhexyl] benzene-1,2-dicarboxylate | 43.59 | 0.35 |  |
| MOL001506 | Supraene | 33.55 | 0.42 |  |
| MOL002879 | Diop | 43.59 | 0.39 |  |
| MOL002897 | epiberberine | 43.09 | 0.78 |  |
| MOL008206 | Moslosooflavone | 44.09 | 0.25 |  |
| MOL010415 | 11,13-Eicosadienoic acid, methyl ester | 39.28 | 0.23 |  |
| MOL012245 | 5,7,4'-trihydroxy-6-methoxyflavanone | 36.63 | 0.27 |  |
| MOL012246 | 5,7,4'-trihydroxy-8-methoxyflavanone | 74.24 | 0.26 |  |
| MOL012266 | rivularin | 37.94 | 0.37 |  |
| MOL001645 | Linoleyl acetate | 42.1 | 0.2 | bupleurum |
| MOL002776 | Baicalin | 40.12 | 0.75 |
| MOL000449 | Stigmasterol | 43.83 | 0.76 |  |
| MOL000354 | isorhamnetin | 49.6 | 0.31 |  |
| MOL000422 | kaempferol | 41.88 | 0.24 |  |
| MOL004598 | 3,5,6,7-tetramethoxy-2-(3,4,5-trimethoxyphenyl)chromone | 31.97 | 0.59 |  |
| MOL004609 | Areapillin | 48.96 | 0.41 |  |
| MOL013187 | Cubebin | 57.13 | 0.64 |  |
| MOL004624 | Longikaurin A | 47.72 | 0.53 |  |
| MOL004628 | Octalupine | 47.82 | 0.28 |  |
| MOL004644 | Sainfuran | 79.91 | 0.23 |  |
| MOL004648 | Troxerutin | 31.6 | 0.28 |  |
| MOL004653 | (+)-Anomalin | 46.06 | 0.66 |  |
| MOL004702 | saikosaponin c_qt | 30.5 | 0.63 |  |
| MOL004718 | α-spinasterol | 42.98 | 0.76 |  |
| MOL000490 | petunidin | 30.05 | 0.31 |  |
| MOL000098 | quercetin | 46.43 | 0.28 |  |
| MOL011680 | N-[(E)-2-[(2R,3S)-3-acetamido-2-(3,4-dihydroxyphenyl)-2,3-dihydro-1,4-benzodioxin-7-yl]vinyl]acetamide | 21.37 | 0.51 | cicada slough |
| MOL011681 | N-[(2S,3R)-7-(2-acetamidoethyl)-3-(3,4-dihydroxyphenyl)-2,3-dihydro-1,4-benzodioxin-2-yl]acetamide | 23.11 | 0.51 |
| MOL011682 | N-[(2R,3S)-7-(2-acetamidoethyl)-2-(3,4-dihydroxyphenyl)-2,3-dihydro-1,4-benzodioxin-3-yl]acetamide | 20.66 | 0.51 |
